# Supplementary material for: Therapeutic itineraries of children after snakebites in the Brazilian Amazon: A thematic drawing-and-story study
Source: PLoS Negl Trop Dis. 2025 Dec 1;19(12):e0013777. doi: 10.1371/journal.pntd.0013777 (PMC12677774; doi:10.1371/journal.pntd.0013777)
Supplement: S1 File — (DOCX) [file pntd.0013777.s001.docx]

**S1 File.** Consolidated Criteria for Reporting Qualitative Research.

COREQ Checklist

Domain 1: Research team and reflexivity

| Item | How addressed in the study |
| --- | --- |
| 1. Interviewer/facilitator | Interviews were conducted by two researchers (J.S.C. and V.A.M.), both experienced in qualitative research. |
| 2. Credentials | MSc-level male researcher with extensive experience in qualitative research and care of SBE patients (J.S.C.), with a male PhD-level qualitative researcher who has a background in Pedagogy and experience in educating children, acting as an observer (V.A.M.). |
| 3. Occupation | Both were researchers affiliated with the Fundação de Medicina Tropical Dr. Heitor Vieira Dourado (FMT-HVD). |
| 4. Gender | Both were male. |
| 5. Experience and training | The interviewers were trained health researchers with expertise in qualitative methods. |
| 6. Relationship established | No prior relationship with the children or their families before recruitment. |
| 7. Participant knowledge of the interviewer | Participants were informed about the study objectives and the role of the researchers. |
| 8. Interviewer characteristics | Researchers emphasized neutrality, used child-friendly approaches, avoided white coats, and adopted clear and informal language. |

Domain 2: Study design

| Item | How addressed in the study |
| --- | --- |
| 9. Methodological orientation and theory | Content analysis was employed, combining inductive coding of the interviews with the theoretical framework of therapeutic itineraries. The drawings were analyzed descriptively according to the topics proposed by Goldner et al. [1], and the itineraries were classified following the model of Cristino et al. [2]. |
| 10. Sampling | Consecutive sampling of children presenting with snakebite during the study period. |
| 11. Method of approach | Children were recruited through notification by the hospital’s multidisciplinary team. Parents were approached first, then children. |
| 12. Sample size | 20 children aged 4–12 years. |
| 13. Non-participation | No dropouts or refusals were reported. |
| 14. Setting of data collection | Interviews were conducted in a quiet, private area of the hospital ward. |
| 15. Presence of non-participants | Parents were present during explanations but interviews prioritized the child’s voice. |
| 16. Description of sample | Children aged 4–12 years, victims of snakebite in the Brazilian Amazon, recruited at FMT-HVD. |
| 17. Interview guide | Semi-structured interviews supported by thematic drawing-and-story procedure (TD-SP). |
| 18. Repeat interviews | No repeat interviews were carried out. |
| 19. Audio/visual recording | Narratives were audio recorded; drawings were collected as part of the data. |
| 20. Field notes | Field notes were taken during and after interviews. |
| 21. Duration | The duration of the interviews lasted approximately 1 hour. |
| 22. Data saturation | Theoretical saturation reached after 17 interviews; 3 additional interviews were conducted to confirm saturation (total n=20). |
| 23. Transcripts returned | Not Applicable. |

Domain 3: Analysis and findings

| Item | How addressed in the study |
| --- | --- |
| 24. Number of data coders | Two independent coders (J.S.C and V.A.M.). |
| 25. Description of the coding tree | A formal coding tree was not developed. Instead, we described the categories and indicators used in the analysis: therapeutic itineraries were classified according to Cristino et al. [2], drawings were assessed descriptively based on style and content indicators adapted from Goldner et al. [1], and interviews underwent inductive content analysis, from which five key themes emerged. These categories and themes are detailed in the Methods and Results sections. |
| 26. Derivation of themes | The themes were derived inductively, from which five key themes emerged. |
| 27. Software | Atlas.ti software (version 7.3.1). |
| 28. Participant checking | Not Applicable. |
| 29. Quotations presented | Children’s verbatim quotes, identified by participant number, were included to illustrate the findings. |
| 30. Data and findings consistent | Findings were consistent with the data presented, triangulated across interviews, drawings, and narratives. |
| 31. Clarity of major themes | Major themes were clearly presented in the results. |
| 32. Clarity of minor themes | Variation in itineraries and less common experiences were also described. |

Developed from Tong et al. [3].

**References**

1. Goldner L, Lev-Wiesel R, Binson B. Perceptions of Child Abuse as Manifested in Drawings and Narratives by Children and Adolescents. Front Psychol. 2021;11. doi:10.3389/fpsyg.2020.562972

2. Cristino JS, de Farias AS, de Melo LDS, Machado VA, Sachett J, Monteiro W. The itinerary of children in search of healthcare: A scoping review and proposal of an explanatory model. Nurs Inq. 2024;31. doi:10.1111/nin.12678

3. Tong A, Sainsbury P, Craig J. Consolidated criteria for reporting qualitative research (COREQ): a 32-item checklist for interviews and focus groups. Int J Quality Health Care. 2007;19(6):349–57.
