# Supplementary material for: Therapeutic itineraries of children after snakebites in the Brazilian Amazon: A thematic drawing-and-story study
Source: PLoS Negl Trop Dis. 2025 Dec 1;19(12):e0013777. doi: 10.1371/journal.pntd.0013777 (PMC12677774; doi:10.1371/journal.pntd.0013777)
Supplement: S2 File — (DOCX) [file pntd.0013777.s002.docx]

**S2 File.** Characterization of the children who were victims of snakebite accidents and participated in the study.

| **Participant** | **Ethnicity** | **Gender** | **Age (years)** | **Education** | **Area of occurrence** | **Time to antivenom (hours)** | **Bite site** | **Severity** | **Local manifestations** | **Systemic manifestations** |
| --- | --- | --- | --- | --- | --- | --- | --- | --- | --- | --- |
| P1 | *Pardo* | Female | 9 | 3rd grade of elementary school | Rural, Iranduba | 1 | Right foot | Mild | Pain, edema, secondary infection | No |
| P2 | *Pardo* | Male | 11 | 5th grade of elementary school | Rural, Iranduba | 1 | Left foot | Dry bite | Mild pain | No |
| P3 | *Pardo* | Female | 12 | 8th grade of elementary school | Urban, Manaus | 1.5 | Left ankle | Mild | Pain, edema | No |
| P4 | *Pardo* | Female | 8 | 3rd grade of elementary school | Peri-urban, Manaus | 1.5 | Right foot | Mild | Pain, edema | No |
| P5 | *Pardo* | Female | 12 | 4th grade of elementary school | Peri-urban, Manaus | 2 | Right ankle | Mild | Pain, edema, secondary infection | No |
| P6 | *Pardo* | Male | 7 | 3rd grade of elementary school | Rural, Manaus | 2 | Left foot | Mild | Pain, edema, secondary infection | No |
| P7 | *Pardo* | Male | 8 | 3rd grade of elementary school | Urban, Manaus | 2.5 | Left foot | Mild | Pain, edema | No |
| P8 | *Pardo* | Male | 11 | 6th grade of elementary school | Rural, Manaus | 3 | Toe – left foot | Mild | Pain, edema | No |
| P9 | Indigenous (Kambeba) | Male | 5 | No | Rural, Manaus | 4 | Toe – left foot | Mild | Pain, edema, secondary infection | No |
| P10 | *Pardo* | Male | 12 | 7th grade of elementary school | Peri-urban, Manaus | 6 | Toe – left foot | Mild | Pain, edema, secondary infection | No |
| P11 | *Pardo* | Female | 6 | 1st grade of elementary school | Rural, Rio Preto da Eva | 1 | Right foot | Mild | Pain, edema, secondary infection | No |
| P12 | *Pardo* | Female | 5 | Preschool | Rural, Rio Preto da Eva | 7 | Left ankle | Mild | Pain, edema | No |
| P13 | *Pardo* | Male | 12 | 7th grade of elementary school | Rural, Manaus | 9 | Right foot | Moderate | Pain, edema, secondary infection | Ecchymosis |
| P14 | Indigenous /Waimiri | Male | 4 | Preschool | Rural, Novo Airão | 12 | Toe – left foot | Mild | Pain, edema, secondary infection | No |
| P15 | Pardo | Male | 4 | Preschool | Rural, Manacapuru | 3 | Left knee | Mild | Pain, edema, secondary infection | No |
| P16 | Pardo | Male | 10 | 4th grade of elementary school | Rural, Iranduba | 20 | Right foot | Mild | Pain, edema, secondary infection | No |
| P17 | Pardo | Male | 11 | 6th grade of elementary school | Rural, Manacapuru | 1.5 | Right ankle | Mild | Pain, edema | No |
| P18 | Indigenous /Apurinã | Male | 10 | Preschool | Rural, Tapauá | 30 | Right ankle and left foot | Severe | Pain, edema, secondary infection, necrosis, compartment syndrome | Epistaxis |
| P19 | Pardo | Female | 9 | 4th grade of elementary school | Rural, Manaus | 32 | Left foot | Moderate | Pain, edema | Hematuria |
| P20 | Indigenous /Waimiri | Male | 9 | 4th grade of elementary school | Rural, Novo Airão | 84 | Right leg | Mild | Pain, edema, secondary infection | No |
