# Supplementary material for: Therapeutic itineraries of children after snakebites in the Brazilian Amazon: A thematic drawing-and-story study
Source: PLoS Negl Trop Dis. 2025 Dec 1;19(12):e0013777. doi: 10.1371/journal.pntd.0013777 (PMC12677774; doi:10.1371/journal.pntd.0013777)
Supplement: S3 File — (DOCX) [file pntd.0013777.s003.docx]

**S3 File.** The environment and participant activities at the time of the snakebite.

| **Participant** | **Environment** | **Quotes from participants** |
| --- | --- | --- |
| P1 | Peridomestic area | *“I went to play right here next to the house. The snake was over there, when I went to pass by the snake to play with my dog, I felt the bite.”* |
| P2 | Workplace^1^ | *“I was coming down from the tree* [the participant was picking fruit] *and I only saw it when it was already biting me, so I quickly took my foot off and ran, crying, to my father.”* |
| P3 | Trail^2^ | *“I was close to home, on the way from the grocery store to my house. I was distracted.”* |
| P4 | Peridomestic area | *“I was on the tree, picking bacuri fruits with my sister. Then, I said I was going to come down, and when I went to walk, I stepped on the thing, and it bit me.”* |
| P5 | Trail | *“I was going to buy bread, on the path we take, which is very close to the bakery. Then, I was playing with my cousin, I didn't see... it was when I turned around, like that, I stepped over the snake.”* |
| P6 | Peridomestic area | *“I came home from school, and I went to wash my feet in the bathroom outside my house, and the snake bit me.”* |
| P7 | Inside house | *"I was playing in my room, with my father lying down on the bed, and my sister was there playing too. He told me to take her pacifier, that she was crying. Then, I didn't even see the snake come in."* |
| P8 | Workplace^3^ | *“I was standing there, helping my father, and then I felt the snake bite me, and when it bit me, I pulled my foot away.”* |
| P9 | Inside house | *“I was playing, playing at the door. It was big and brown, it bit me.”* |
| P10 | Peridomestic area | *“I was playing soccer, then I went home and got some water. Then, I went to the backyard where my stepfather was burning some bushes. I was going to burn the bushes, but when I reached the bushes, it bit me.”* |
| P11 | Peridomestic area | *“I was alone, my foot hurt. I looked at the ground, it was curled up.”* |
| P12 | Trail | *“I was going back to grandma's house, with auntie, when it bit me and I didn't even see it.”* |
| P13 | Recreation area^4^ | *“I went to change my clothes, like this, in the woods, next to a little road. Then, when I was already leaving the woods, an animal bit my leg, and all I saw was bleeding.”* |
| P14 | Workplace | *“He doesn’t remember well, he just kept crying. Then, his mother ran to see him and called me. When I saw him, I said that’s not an ant, it’s a snake.”* (translated by the father from the indigenous language) |
| P15 | Peridomestic area | *“I was with grandma, picking peppers. I just felt pain, so I yelled to her.”* |
| P16 | Trail | *“I went to my friend’s house, it was morning. When I was going home, on the trail, with my uncle behind and my father in front, I was in the middle. I was feeling fine, then I felt something sting me. I said: Dad, something bit me! Then, I started to cry.”* |
| P17 | Peridomestic area | *“I was picking olives, it was close to home, it bit me and I immediately called dad.”* |
| P18 | Workplace^5^ | *“He was looking for pineapple, right? At that time, he was in the fields. He passed by a fallen tree trunk, right? That’s when the snake bit him.”*  (translated by the mother from the indigenous language) |
| P19 | Peridomestic area | *“I went to throw an old cloth, which I had used for cleaning at home, and then I just felt the bite, but I didn't see what bit me.”* |
| P20 | Workplace^6^ | *“In the morning, he went fishing. Then, he was supposed to leave at 8 am, and when they arrived at the fishing spot, the snake bit him.”*  (translated by the uncle from the indigenous language) |

^1^ Collecting fruit and helping his father with his fishing work.

^2^ Narrow pedestrian paths between two or more residences or between residences and small shops in rural areas.

^3^ Playing and Helping father at work.

^4^ Near a river beach.

^5^ Collecting fruit and helping mother on the farm.

^6^Helping his brother with his fishing work.
