# Supplementary material for: Therapeutic itineraries of children after snakebites in the Brazilian Amazon: A thematic drawing-and-story study
Source: PLoS Negl Trop Dis. 2025 Dec 1;19(12):e0013777. doi: 10.1371/journal.pntd.0013777 (PMC12677774; doi:10.1371/journal.pntd.0013777)
Supplement: S4 File — (DOCX) [file pntd.0013777.s004.docx]

**S4 File.** Use of the popular healthcare sector in the therapeutic itineraries of the study participants.

| **Participant** | **Pre-hospital popular treatment** |
| --- | --- |
| P1 | Washed the bite site with an infusion of alcohol and herbs, and kept the leg elevated |
| P2 | Washed the bite site with water |
| P3 | Performed a tourniquet |
| P4 | Washed the bite site with water, and applied anointing oil^1^, ice and alcohol to the bite site |
| P5 | Massaged and squeezed the bite site^2^ |
| P6 | Washed the bite site with water and performed a tourniquet |
| P7 | None |
| P8 | Wash the bite site with soap powder and alcohol |
| P9 | None |
| P10 | Washed the bite site with water, and took metamizole |
| P11 | Washed the bite site with water |
| P12 | Performed a tourniquet |
| P13 | Washed the bite site with bar of soap, running water and lemon, and squeezed the area^2^ |
| P14 | Drank juice from a thorny plant^3^, and applied juice from that same plant to the bite site |
| P15 | Washed the bite site with water, soap and vinegar, and took metamizole and *Específico Pessoa*^4^ |
| P16 | Took metamizole and performed a tourniquet |
| P17 | Kept (the child and his father) the snakebite a secret until he arrived at the hospital^5^ |
| P18 | Washed the bite site with water |
| P19 | Washed the bite site with water, and took metamizole and *Torsilax*^6^ |
| P20 | Took metamizole and antihistamines |

^1^ Oil traditionally used in religious rituals, blessed by a religious leader, for anointings generally accompanied by prayers, for the purpose of protection or treatment of illnesses. It is common for people to purchase and keep little containers of this oil in their homes.

2 Exerting pressure around the fang marks or squeezing the bitten limb to promote bleeding and 'removal of the snake venom'.

3 With only this information, it was not possible to determine the plant species.

4 Preparation of unknown composition easily found in stores in the Amazon region, especially in agricultural product stores, popularly used to treat snakebite in humans and animals.

5 Strategy to avoid ‘evil eye’, the popular name for the so-called negative energies transmitted by someone to another person, hindering the realization of plans or achievements at some level.

6 Over-the-counter medicine with caffeine, carisoprodol, diclofenac and paracetamol.
