# Supplementary material for: Therapeutic itineraries of children after snakebites in the Brazilian Amazon: A thematic drawing-and-story study
Source: PLoS Negl Trop Dis. 2025 Dec 1;19(12):e0013777. doi: 10.1371/journal.pntd.0013777 (PMC12677774; doi:10.1371/journal.pntd.0013777)
Supplement: S5 File — (DOCX) [file pntd.0013777.s005.docx]

**S5 File**. Children’s Drawings Depicting Snakebite Envenomation: A Descriptive Analysis

**Participant 2**


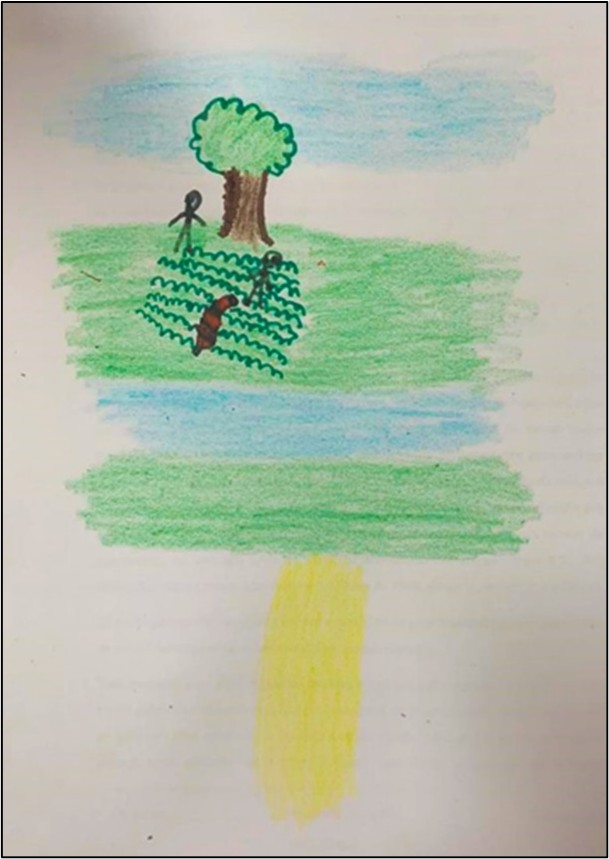


The drawing portrays the snakebite accident experienced by an 11-year-old male child, a 5th-grade elementary school student. The incident occurred in an occupational setting, while the child was accompanying his father during fruit collection from a tree. After being bitten by a snake, the boy was taken by motorcycle to his home. From there, he walked with his mother to a primary health care unit in Iranduba/AM, a municipality in the state of Amazonas, from where he was transferred by ambulance to the Fundação de Medicina Tropical Dr. Heitor Vieira Dourado (FMT/HVD) in Manaus.

The drawing made by the child presents a single and detailed scene of the accident, which occurred during the day. Elements of the place are shown, such as the tree where they were collecting fruits, the surrounding vegetation, and the river that the boy had to cross to reach the road leading to his house. The child represents himself next to his father, showing their closeness at the time of the incident. The snake is positioned among the vegetation, also at the center of the scene, with an exaggerated size compared to the other elements.

The graphic style of the drawing is classified as realistic, appropriate for the age of 11 years, with the use of proportions, an attempt at perspective, and fidelity to the colors and shapes of the natural environment. The victim is represented proportionally, positioned at the center of the composition, which suggests the centrality of the event in the child’s memory. The snake also occupies the center of the scene.

The drawing provides a description of the environment and the people present at the time of the accident, such as the father, the river, and the vegetation, but does not include the events after the accident, the means of transportation used, the hospital, or the medical care received. Nor is there representation of pain, injury, or physical symptoms, although contact with the snake is clearly suggested by the proximity depicted in the drawing. There is no presence of multiple scenes or movement; the narrative focus is concentrated on the location and the exact moment of the accident.

**Participant 3**


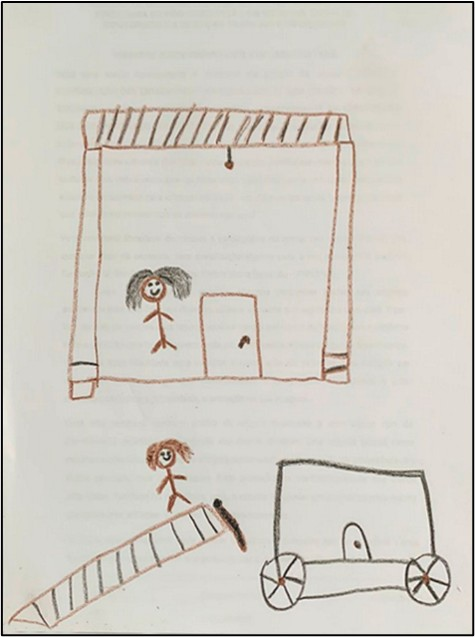


The drawing portrays the snakebite accident experienced by a 12-year-old female child, an 8th-grade elementary school student. The incident occurred in the peridomicile area, specifically on a staircase located on the way back to her home. After the bite, the girl was taken by her mother in a neighbor’s car to an emergency hospital, which, however, was unable to treat the case. From that unit, the child was instructed to proceed to the Fundação de Medicina Tropical Dr. Heitor Vieira Dourado (FMT/HVD), still in the same vehicle, in the city of Manaus/AM.

The drawing made by the child represents a single scene, focused on the moment of the accident. The staircase where the bite occurred is well characterized, with the snake positioned on the ground, among the steps. The girl is also depicted near the staircase and in proportional size, while the snake appears disproportionately large. Next to the staircase, the child’s house is drawn, with the figure of the mother visible inside, awaiting her return. The neighbor’s car, which served as the means of transportation to the hospital, is also included in the scene.

Although the drawing does not present multiple scenes nor convey movement, it organizes well the spatial and relational elements of the experienced moment. The style is classified as schematic, which is atypical for the child’s chronological age, considering that at 12 years old, a more realistic style would be expected. Even so, the drawing is coherent in its symbolic construction, with recognizable elements and a clear visual narrative.

The snake is represented in a peripheral position but close to the victim, suggesting visual and physical contact. The means of transportation and the presence of the mother as a companion are included; however, the hospital, the pain, the injury, and the medical care are not illustrated. Words were not included in the drawing.

**Participant 5**

**
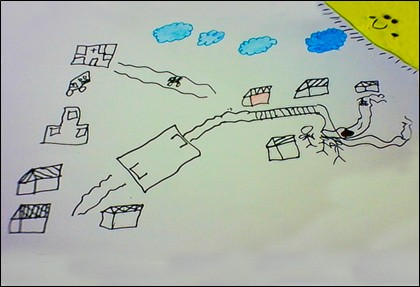
**

The drawing portrays the snakebite accident experienced by a 12-year-old female child, a 4th-grade elementary school student. The incident occurred in the peridomicile area, while the girl was on her way to the bakery with her 7-year-old cousin, leaving from home. Along the way, there were two possible routes, and both chose the shorter one, which passed through a neighbor’s plot of land with dense vegetation—a feature that made it difficult to see the ground and consequently contributed to the accident. After being bitten, the child was carried by her cousin to the neighbor’s house and from there transported by motorcycle taxi to the hospital closest to her residence. Subsequently, she was transferred by ambulance to the Fundação de Medicina Tropical Dr. Heitor Vieira Dourado (FMT/HVD).

The drawing made by the child sequentially represents the main elements of the accident and the journey to medical care. The composition suggests more than one scene, showing both the environment of the accident and the means of transportation used. The girl highlights the two available paths, pointing out the chosen route with the presence of the snake, drawn in a circular black shape. She also depicts the nearby soccer field, as well as the neighborhood houses. The motorcycle used to take her to the nearest hospital and the ambulance that transported her to the FMT/HVD are also represented.

The scene takes place during the day, as indicated by the clear sky and the presence of the sun in the drawing. The snake aggressor is represented proportionally and in a peripheral position, close to the victim, suggesting the moment of the bite. The child also draws herself in proportional size, positioned peripherally in the composition. The cousin and her mother are represented at her side in the drawing. A black spherical dot is noticeable, representing the coiled snake next to her in the drawing.

The drawing style is realistic, appropriate for the age group, with correct proportions, an attempt at perspective, the use of shading, and colors close to reality. The drawing conveys a sense of movement and displacement, with scenes that represent the dynamism of the event.

Although the drawing represents the environment, the vehicles used, the hospitals, and the route taken, there is no explicit representation of pain, the lesion caused by the bite, health professionals, or the medical care received.

**Participant 7**


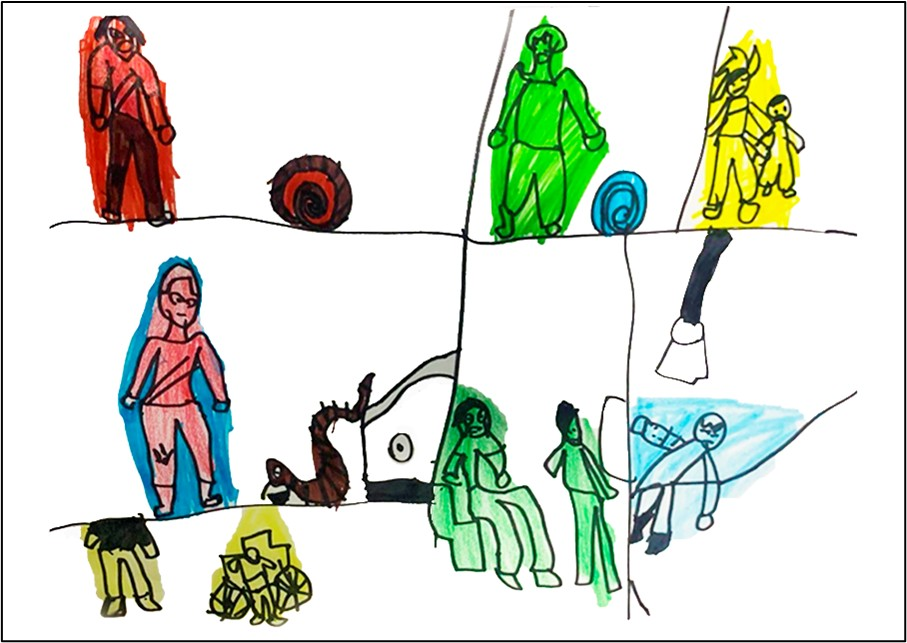


The drawing portrays the snakebite accident experienced by an 8-year-old male child, a 3rd-grade elementary school student. The incident occurred at home, specifically in the laundry area. After the accident, the boy was rescued by his father, who took him in his car/bus to a children’s emergency department in the city of Manaus (AM). Later, he was transported in his aunt’s car to the Fundação de Medicina Tropical Dr. Heitor Vieira Dourado (FMT/HVD), where he received specialized care.

The drawing made by the child is presented in a comic-strip format, with multiple panels that sequentially depict the main moments of the accident and the journey to hospital care. In the first panel, shaded in red, the child draws himself wearing a school uniform, approaching a coiled snake on the ground. In the second, the snake bites the boy’s leg. In the third panel, the child appears being rescued by his father, who heard his cries. In the fourth, he is drawn again next to the snake, now represented beside a washing machine, reinforcing the exact location where the accident occurred; the snakebite marks on his right leg are also shown. In the fifth panel, he is inside his father’s bus, on the way to the hospital. In the sixth, he is already lying in the hospital with an IV stand beside him. Finally, in the last panel, he appears inside a second car, representing the transfer to the FMT/HVD.

The drawing style is classified as schematic, consistent with his age group, with the use of recognizable shapes, varied colors, spatial organization, and a well-defined narrative sequence. The child is represented in a central position, with proportional size. The snake aggressor also occupies a central position and is drawn in an exaggerated size.

The drawing includes elements that indicate movement, the route taken, the means of transportation used, the presence of companions, and the representation of the hospital. Clear signs of pain are present, such as facial expressions of crying and sadness, along with the depiction of the local injury, with visible blood on one leg in one of the scenes. Although there is no representation of systemic lesions, hospital care is illustrated, including the IV fluid and the resting posture on the hospital bed.

**Participant 8**


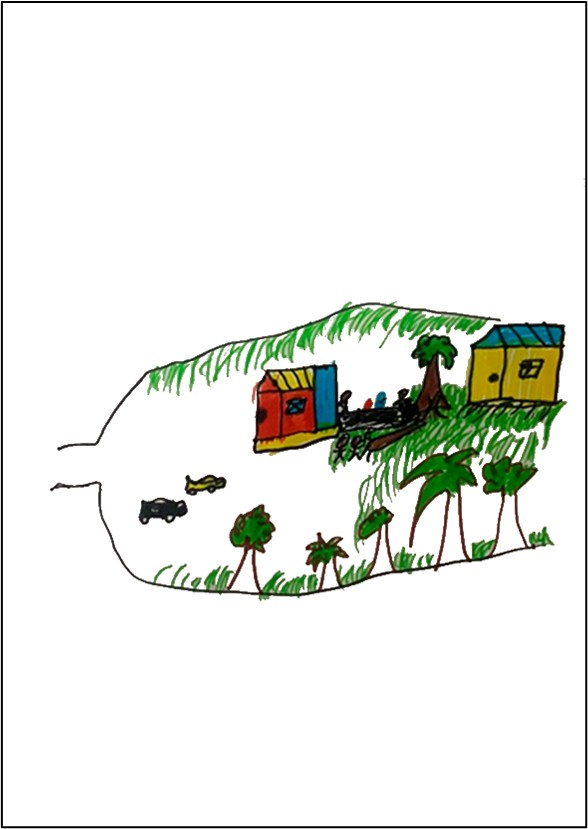


The drawing portrays the snakebite accident experienced by an 11-year-old male child, a 6th-grade elementary school student. The incident occurred in an occupational context, in the yard of a small farm where the child accompanied his father, who was working on the renovation of a house. While playing nearby, the child was bitten by a snake. After the accident, he was taken by his father in his car to their residence, located in another neighborhood. During the journey, they stopped to pick up the child’s mother, and together they went directly to the Fundação de Medicina Tropical Dr. Heitor Vieira Dourado (FMT/HVD) in Manaus.

The drawing made by the child depicts in detail the natural and family environment of the small farm where the accident occurred. The setting is predominantly rural, with dense vegetation and many trees, suggesting a remote and natural place. At the center of the composition are two houses: one red with yellow details and another yellow with blue details. The first represents the house under renovation, where the father was working, and the second represents another existing residence at the site. Near the house under renovation, the child draws himself playing, in small size, and human figures represent the father and other people involved in the work.

Although the aggressor snake is not depicted, the context of the scene is well defined. In the lower left part of the drawing, cars appear symbolizing the means of transportation used to seek help, even though the full route to the hospital and the sequence of events are not visually represented. The drawing uses vivid colors and spatial organization, composing a narrative focused on the moment of the accident and the environment where it occurred, without multiple scenes or a sense of movement.

The graphic style is realistic, appropriate for the child’s age group, with an attempt at perspective, coherent proportions, textures, and discreet emotional elements. The child is represented at the center of the scene, in small size compared to the other elements. The hospital, the snake, the injury, and the pain are not illustrated, nor is the medical care received.

**Participant 9**


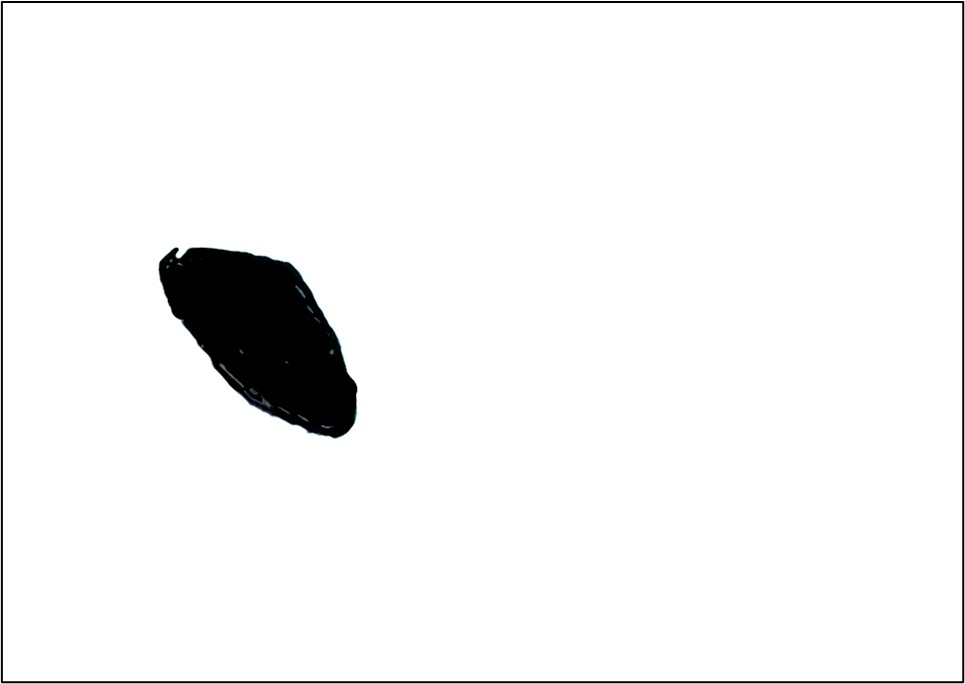


The drawing portrays the snakebite accident experienced by a 5-year-old male child, Indigenous, non-literate, and not attending school. The accident occurred at his home, while the child was playing indoors, near the entrance door. After the incident, the child was carried by his father to the base health post of his community and from there was transferred by helicopter to Manaus, where an ambulance was already waiting to take him to the Fundação de Medicina Tropical Dr. Heitor Vieira Dourado (FMT/HVD), where he received specialized care.

The drawing produced by the child consists of a scribble, typical traces of the first stage of graphic development in childhood and, according to his verbal account, represents the place of the accident—his house. Although the traces are still rudimentary, the child indicates with intention that the figure drawn symbolizes his residence, demonstrating an attempt to express himself through the cognitive and motor resources available to him.

There is no representation of the participant (the child himself), of the aggressor snake, of the injury, or of the means of transportation used. Neither are the route to the hospital, the pain, the medical care received, nor the presence of companions represented. The drawing does not present multiple scenes nor convey movement, being limited to the accident environment.

The graphic style is consistent with his age group and stage of development and reflects the controlled scribble stage, in which the traces already have some intentionality, although they are not yet recognizable by external observers without the child’s verbal mediation. The absence of detailed elements and the limitation of visual content do not represent a lack of experience of the event but rather reflect the expressive capacities of a young, non-literate child in the initial process of symbolic development.

**Participant 10**


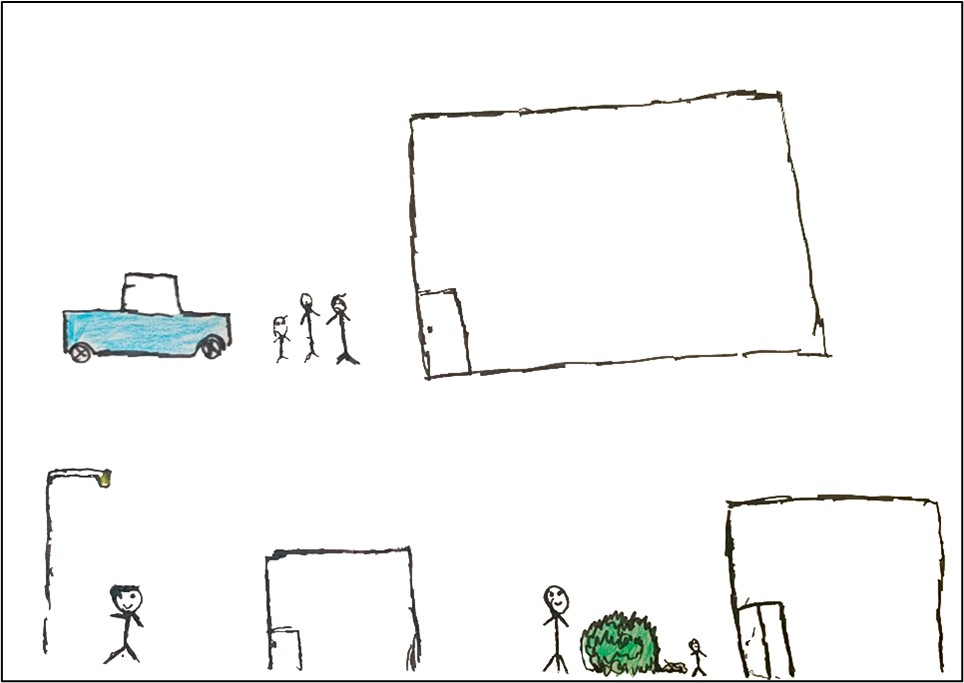


The drawing portrays the snakebite accident experienced by a 12-year-old male child, a 7th-grade elementary school student. The incident occurred in the peridomicile area, while the child was helping his stepfather burn branches and brush in the backyard of their house. During this activity, he was bitten by a snake hidden among the vegetation. After the accident, he was taken by car to an emergency unit and later, also in a private vehicle, to the Fundação de Medicina Tropical Dr. Heitor Vieira Dourado (FMT/HVD) in Manaus (AM), where he received specialized medical care.

The drawing made by the child presents an organized narrative sequence, distributed vertically, from the bottom to the top of the page. This structure shows the events in chronological order, from the accident site to the arrival at the hospital. In the lower right corner, the exact location of the accident is depicted, with the house in a rectangular shape, the boy and his stepfather near the green branches to be burned, and the black snake on the ground, partially hidden, next to the vegetation.

On the left side of the lower part, the child draws a nighttime scene, with a lit lamppost, representing the moment when he waited outside his house for transportation. This element reinforces the nighttime setting of the accident. In the upper part of the drawing, the narrative is completed with the representation of the hospital, a large rectangle with a visible door, indicating the child’s arrival, accompanied by his mother and stepfather, at the healthcare facility in their own car.

The drawing is composed of schematic strokes, a style consistent with the child’s age group. The spatial organization is well defined, with good proportions among the elements. The boy depicts himself in a central position and in proportional size, while the snake appears in a peripheral position and in small size.

The drawing represents the route taken, the means of transportation used, the companions, and the hospital. However, there are no graphic signs of pain, visible lesions, systemic manifestations, or direct representation of medical care. No words are written in the drawing.

**Participant 11**


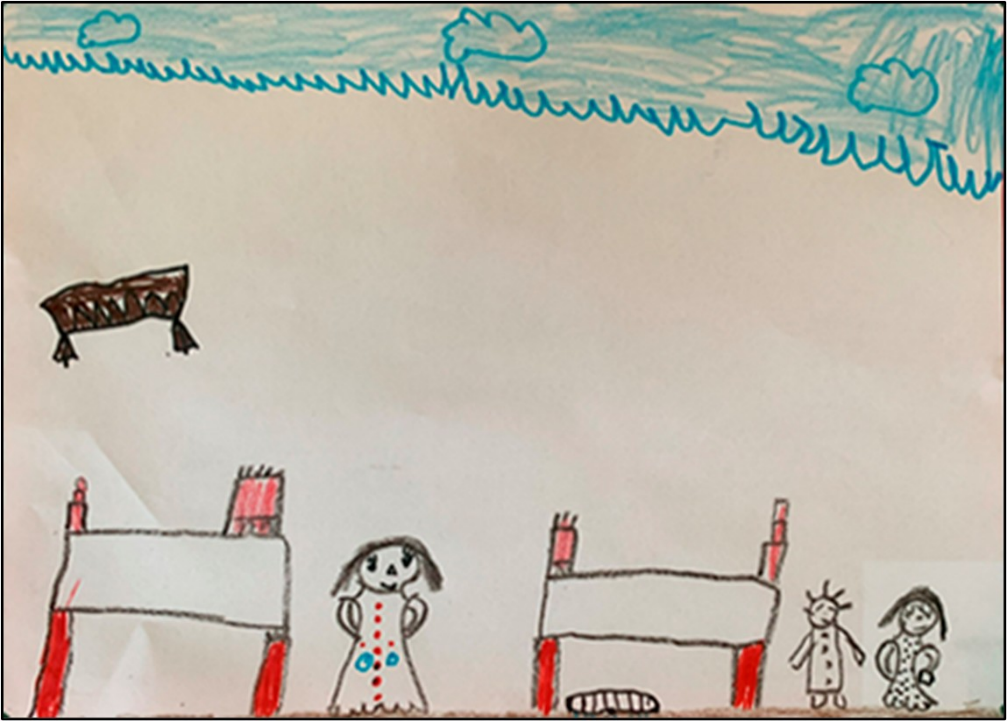


The drawing portrays the snakebite accident experienced by a 6-year-old female child, a 1st-grade elementary school student. The incident occurred in the peridomicile area, while the girl was in the backyard of her house, near a small wood stove. After the accident, she was rescued by her father, who took her by motorcycle to the hospital in the municipality of Rio Preto da Eva (AM). She was then transferred by ambulance to the Fundação de Medicina Tropical Dr. Heitor Vieira Dourado (FMT/HVD) in Manaus (AM).

The drawing made by the child represents the moment of the accident, which occurred during the day, as indicated by the presence of a blue sky with clouds. At the center of the composition, the girl draws herself next to the stove, represented with red colors and a brown upper figure symbolizing pieces of wood. Beside this scene, the stove reappears, this time with the snake positioned underneath it, indicating the exact place of the attack and where the snake was at the moment of the accident.

In addition to the victim and the aggressor animal, the drawing includes two additional characters: the child’s brother and sister, shown at a distance observing the scene. The presence of these witnesses reinforces the reconstruction of the accident moment and the family context in which it occurred. The snake is represented proportionally, in a central position, as is the child herself.

The drawing style is classified as schematic, appropriate for her age group. The elements are arranged in an organized manner, with the use of vivid colors, recognizable shapes, and an attempt to compose a complete setting. The child depicts herself proportionally, at the center of the scene, in close proximity to the snake. Although the drawing does not present movement or clinical developments such as pain, injuries, or the hospital, it clearly conveys the environment and the moment of the attack. The route to the hospital, the means of transportation, and the medical care are not represented, and no written words appear in the drawing.

**Participant 12**


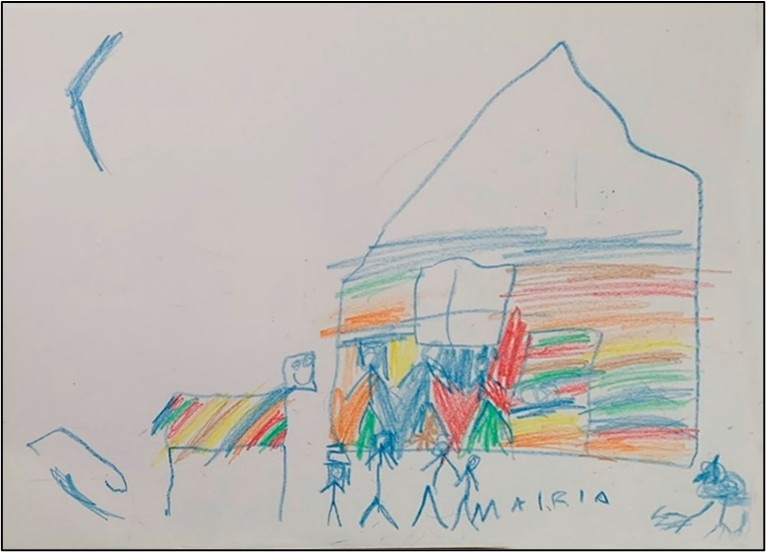


The drawing portrays the snakebite accident experienced by a 5-year-old female child, a preschool student in the 2nd year. The incident occurred in the peridomicile area, specifically in the backyard of her grandmother’s house, while the child was feeding the chickens. After the bite, she was taken in a private car, accompanied by her mother, to the hospital in the municipality of Rio Preto da Eva (AM), where there was no adequate care for the case. She was then taken, still in the same vehicle, to the Fundação de Medicina Tropical Dr. Heitor Vieira Dourado (FMT/HVD) in Manaus.

The drawing produced by the child consists of strokes and shapes typical of the pre-schematic stage, with the use of various colors. The scene depicted takes place at night, as indicated by visual elements such as the dark sky and the representation of the moon. The girl represents her grandparents’ house with human figures visible inside the residence, showing her attention to the environment and family members present at the time of the accident. She also draws the family dog and one of the chickens that were present during the snakebite accident, as well as all the people who accompanied her in the activity of feeding the chickens, namely her grandmother and aunts.

The child draws herself next to the family dog and in exaggerated size. However, the aggressor snake, the injury, and the contact with the animal are not represented. There are no signs of movement, clinical signs and symptoms, or hospital representation, but there are words in the drawing in which the child signs her name.

The drawing corresponds to what is expected for her age and schooling stage, with recognizable forms, the use of various colors, and spatial organization. Despite the absence of the snake, details of the accident environment are noticeable, such as the presence of family members, animals, the house, and the night sky.

**Participant 13**


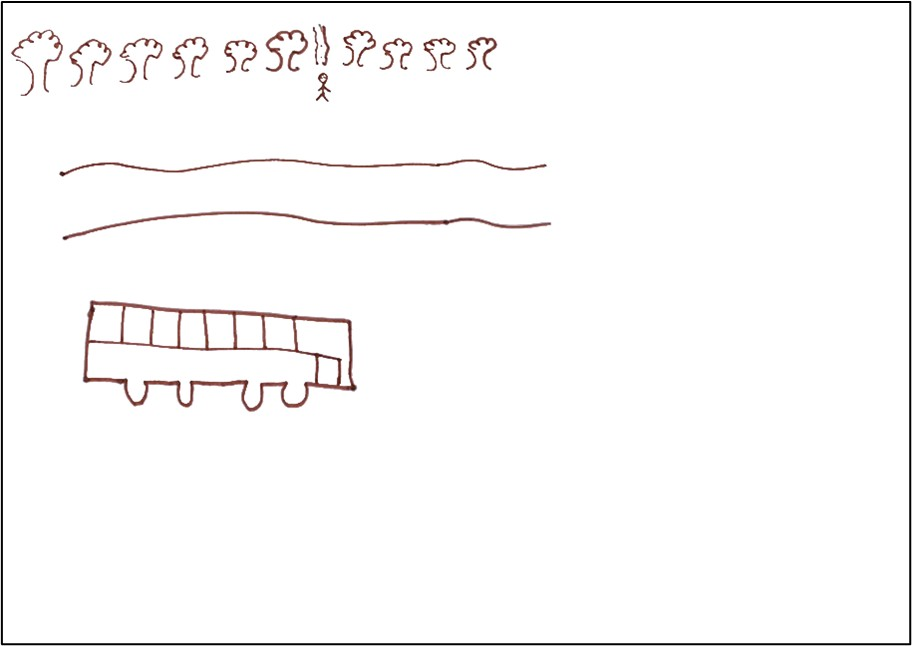


The drawing portrays the snakebite accident experienced by a 12-year-old male child, a 7th-grade elementary school student. The incident occurred in a leisure setting, near a bathing area, while the boy moved away from the group to urinate behind a tree on the trail. After being bitten, he returned home by bus. Later, he was taken by relatives in a car to a Children’s Emergency Department in the city of Manaus (AM). Still without definitive care, he went to an Emergency Care Unit (UPA), where he received guidance and was referred to the Fundação de Medicina Tropical Dr. Heitor Vieira Dourado (FMT/HVD), also in a private vehicle throughout this journey.

The drawing produced by the child represents, in a simple and direct way, the environment where the accident occurred, with emphasis on the vegetation of the site. At the top of the drawing, a row of trees symbolizes the forest near the bathing area. In the center of the image, the child draws himself leaving a trail that crosses the vegetation, indicating the place where the bite occurred. Below this natural area, there is a road, and at the bottom of the drawing, a bus is represented in profile, with details such as wheels and windows, referencing the transportation used to return home after the accident.

The aggressor snake, the hospital, and the companions are not represented, nor are scenes of pain, injury, or medical care. Although the full route after the accident is not depicted, the drawing includes one of the means of transportation used, as well as the accident environment. The child also draws himself in a peripheral position and in small size.

The drawing style is classified as schematic, although the age and school level of the child would suggest an expectation of a more realistic style. Even so, the use of basic symbols and coherent spatial arrangement demonstrates an effort of visual communication and organization of the event.

**Participant 14**


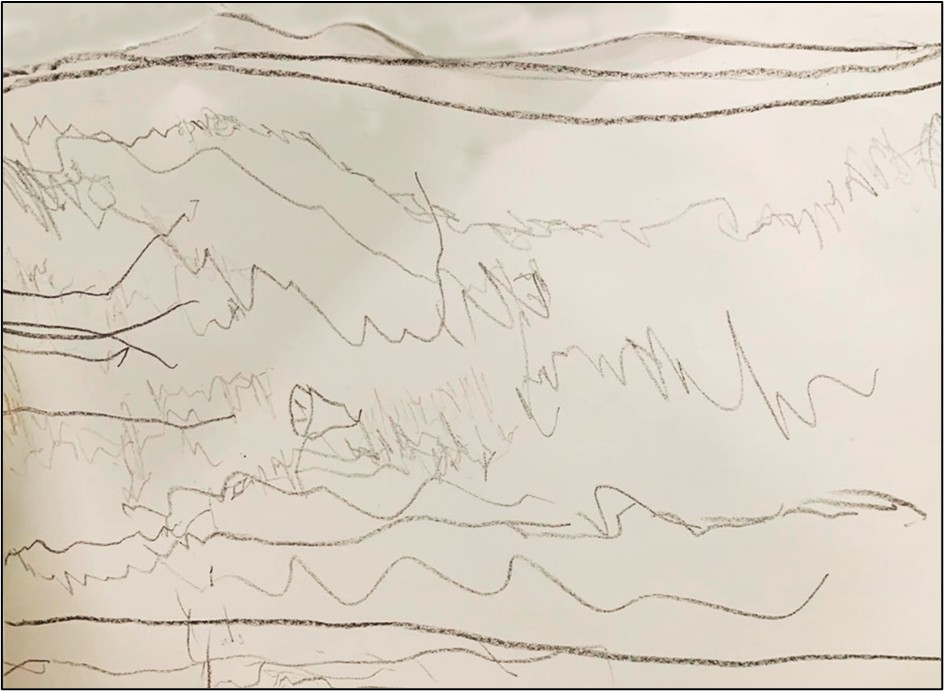


The drawing portrays the snakebite accident experienced by a 4-year-old male Indigenous child, a preschool student in the 1st year. The incident occurred in an occupational context, while the child was playing in the fields accompanied by his parents. After the accident, the father took him in a small motorized canoe (voadeira) to their house in the village. Then, still in the same boat, they went to a larger village, from where they were sent, in another canoe, to the base health post. From there, the child was once again transported by canoe to the port of the municipality of Novo Airão (AM), where an ambulance was waiting to take them to the municipal hospital. Subsequently, the child was transferred, also by ambulance, to the Fundação de Medicina Tropical Dr. Heitor Vieira Dourado (FMT/HVD) in Manaus (AM).

The drawing produced by the child consists of scribbles, typical of the early stage of graphic development, especially in young children. The image does not attempt to depict the moment of the accident itself but rather shows the child’s attempt to represent the river journey in search of medical care. At the center of the drawing, a set of lines appears to represent the child inside the canoe, while marks around it seem to symbolize the choppy river water. At the edges of the page, more concentrated strokes indicate the riverbanks, completing the setting and contextualizing the journey experienced.

The child does not represent himself in an identifiable way, nor are there depictions of companions, the aggressor snake, injuries, hospital, or medical care. There is also no presence of multiple scenes or other narrative elements. The graphic style corresponds to the controlled scribble stage, in which the traces, although still rudimentary, carry intentionality. The child uses the resources available to symbolize aspects of the experience lived.

**Participant 15**


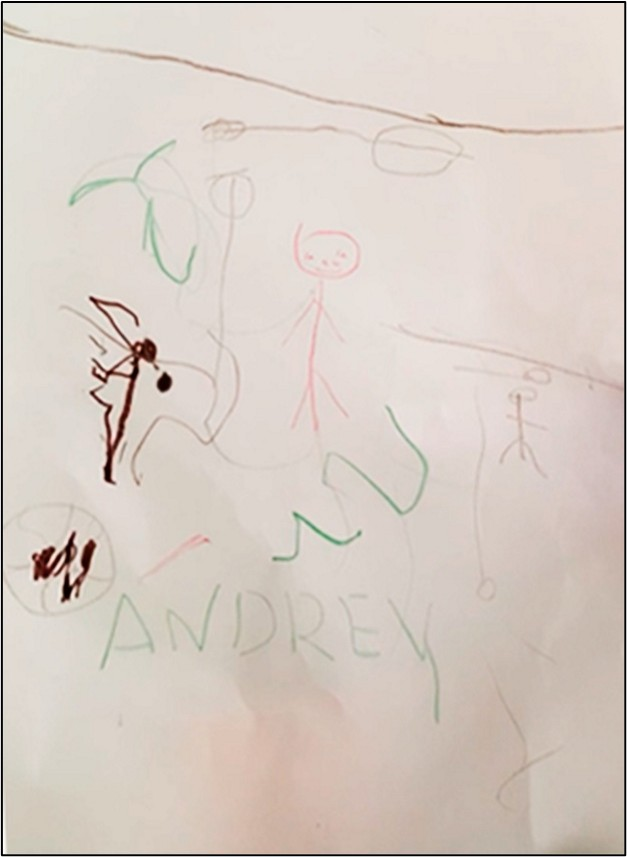


The drawing portrays the snakebite accident experienced by a 4-year-old male child, a preschool student in the 1st year. The incident occurred in the peridomicile area, while the boy was returning home with his grandmother after both had gone to pick peppers. During this journey, he was bitten by a snake. After the accident, the child managed to return home on foot and then walked to the residence of a community health worker. From there, he went to the port, where he boarded a boat bound for the municipality of Manacapuru (AM). From the port, he was taken in a private car to the municipal hospital and, after medical evaluation, was transferred by ambulance to the Fundação de Medicina Tropical Dr. Heitor Vieira Dourado (FMT/HVD) in Manaus (AM), where he received specialized care.

The drawing produced by the child consists of simple strokes, characteristic of the pre-schematic stage, typical for the age of 4. The composition presents scribbles and basic shapes, with free use of colors. At the center of the image, the boy depicts himself next to a figure symbolizing his grandmother, indicating who was with him at the time of the accident. Nearby, in a dark red line, he represents the snake, the animal responsible for the accident, in a peripheral position. The child signs his name on the drawing, located at the bottom of the page.

The graphic style demonstrates an early stage of children’s drawing development, with symbolic, disproportionate representations and still poorly organized spatially. The figure of the child appears in exaggerated size, as does the snake. The scene is centered on the moment of the accident, without representation of movement, the route, or the medical care received. Although the child experienced a long journey before receiving hospital care, this part was not included in the drawing.

The drawing includes the author and the aggressor snake, but there is no illustration of pain, the bite lesion, systemic symptoms, or the hospitals. Nor are there visual elements representing the means of transportation used, although the grandmother, who was present at the time of the accident, is included. The drawing presents a single static scene, centered on the moment of the encounter with the animal.

**Participant 18**


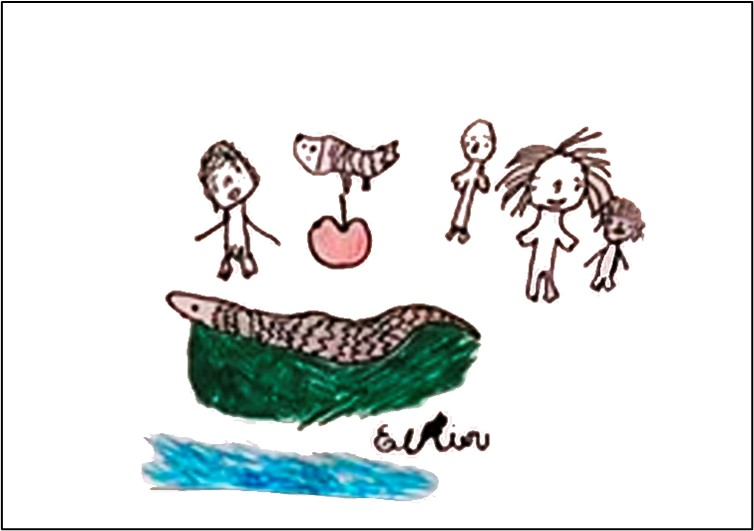


The drawing portrays the snakebite accident experienced by a 10-year-old male child, a 1st-grade elementary school student. The incident occurred in an occupational context, while the child was helping his mother and siblings in the fields. During the activity of harvesting a pineapple, he was bitten by a snake. After the accident, he was taken by canoe to the village, by motorboat to the base health post, and then transported by ambulance to the municipal hospital of Tapauá, a municipality in the state of Amazonas, where he received antivenom. Subsequently, he was transferred by airplane to the referral hospital in the state capital, Manaus.

The drawing made by the child prominently depicts the farming environment where the accident occurred. At the center of the image, the aggressor snake appears in a highlighted position, close to the boy, suggesting visual and physical contact. The child also draws himself in the scene, in a central position, with exaggerated size compared to the other elements. Nearby are his family members—his mother and siblings—reinforcing the collective context of the activity being carried out. In the background, a domestic dog is visible, and in the middle of the illustration, the boy represents what appears to be the pineapple he was harvesting at the time of the accident.

The natural environment is complemented by the representation of the river and surrounding vegetation, reinforcing the geographic and environmental characteristics of the accident site. Despite the richness of descriptive elements, the drawing does not present multiple scenes nor convey a sense of movement or displacement. The focus is centered on the moment of the accident, without representation of the journey to the hospital, the means of transportation used, or the medical care received.

The drawing style is classified as schematic, consistent with the child’s age and schooling, even though he is still in the early stages of literacy. The strokes are organized, with recognizable shapes and coherent spatial arrangement. Both the snake and the victim appear in exaggerated size. The drawing includes written words, specifically the child’s name as a signature. It also represents the presence of companions at the time of the accident, but there are no signs of pain, injury, clinical manifestations, or the presence of healthcare professionals.

**Participant 19**


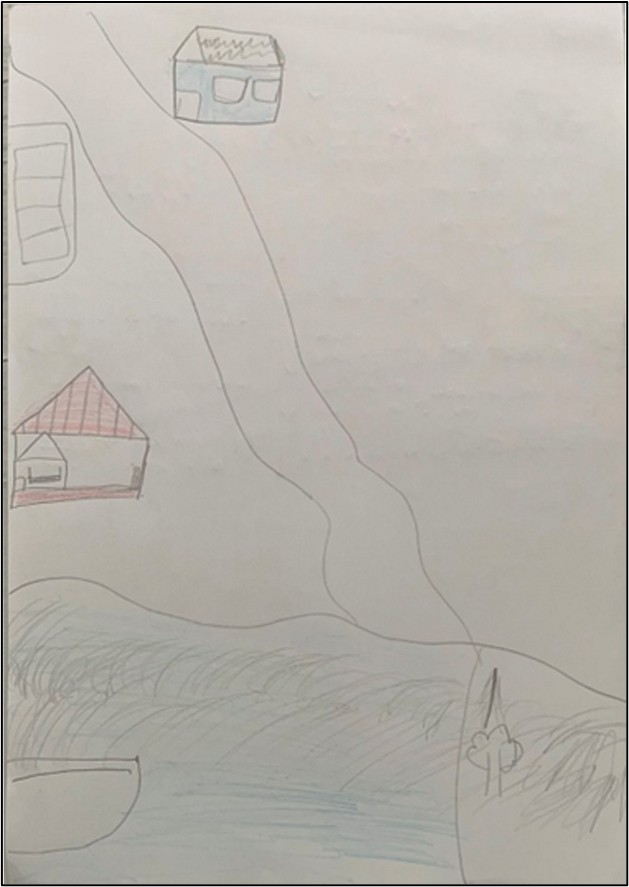

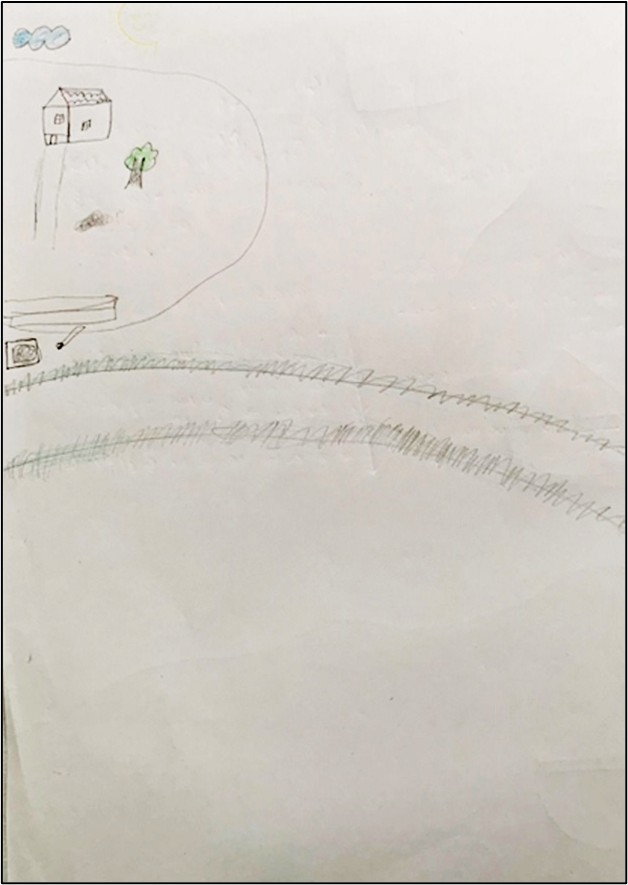


The drawing portrays the snakebite accident experienced by a 9-year-old female child, a 4th-grade elementary school student. The incident occurred in the peridomicile area, in the backyard of her house, while the girl was taking out the trash. Only on the day following the accident was she taken by motorcycle to a health post near her residence. From there, she continued on foot to the port, where she crossed the river by voadeira (small motorized canoe). She was then transported by boat to a second port in the city of Manaus (AM). Upon disembarking, she went by car to a Children’s Emergency Department, and was later transferred by ambulance to the Fundação de Medicina Tropical Dr. Heitor Vieira Dourado (FMT/HVD), also in Manaus (AM).

The child produced two sequential drawings, organized as a visual narrative of the accident and part of the journey to care. The first drawing depicts the accident site. The child’s house appears at the top of the page, next to a tree representing the natural environment of the backyard. A river cuts through the middle of the composition, indicating the route that would be taken the following day toward medical care. Near the riverbank, the port from which the voadeira departed is represented. The scene takes place during the day, as indicated by the blue clouds and yellow sun in the sky.

The second drawing focuses on the urban setting upon arrival in Manaus. In it, the river is represented again, with part of the boat still visible in the water. A road appears, connecting houses arranged along the way, reinforcing the transition from the rural to the urban environment. Both drawings are organized coherently and clearly, presenting more than one scene and evidence of displacement.

Although the child does not depict herself in the images, the means of transportation used, such as the boat and the road with cars, are symbolically present. The aggressor snake, the injury, the companions, and the hospital are not illustrated, nor are pain or the medical care received. The graphic style is classified as schematic, consistent with the child’s age, with organized strokes, recognizable elements, and the use of colors close to reality.

**Participant 20**


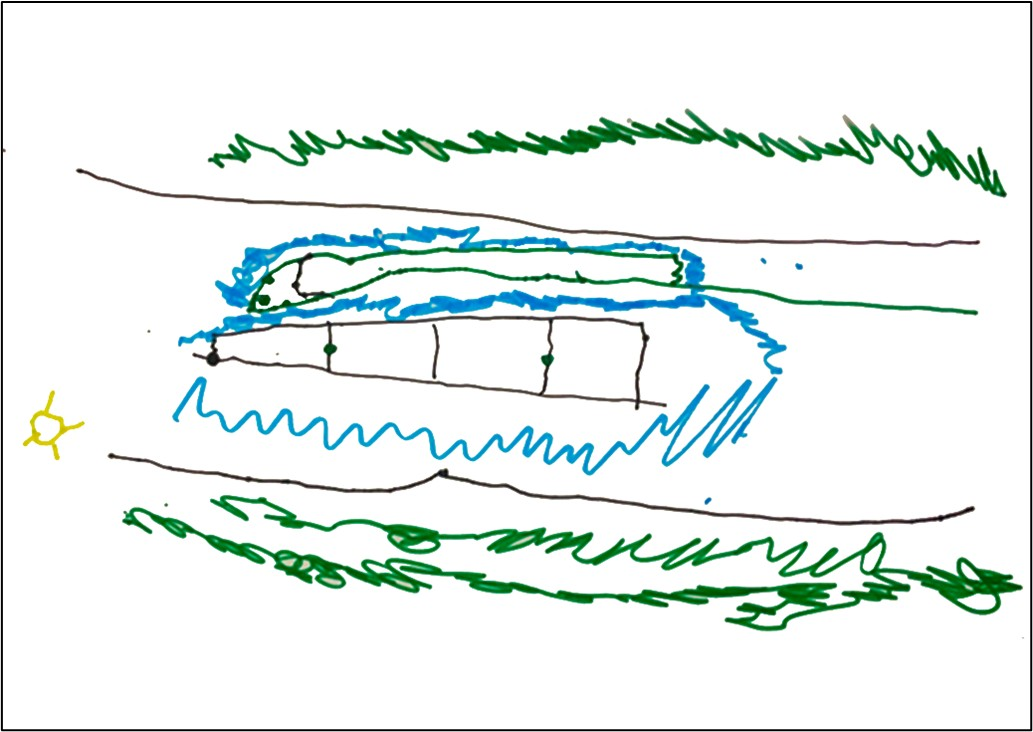


The drawing portrays the snakebite accident experienced by a 9-year-old Indigenous male child, a 4th-grade elementary school student. The incident occurred in an occupational context, while the child was fishing in the river in a canoe, accompanied by his older brother. After the bite, they returned to the village by canoe. The child was then transported by voadeira (small motorized canoe) to the base health post and, later, by boat to the port of the municipality of Novo Airão, in Amazonas, where an ambulance was already waiting to take him to the municipal hospital. After a long waiting period, he was transferred by ambulance to the Fundação de Medicina Tropical Dr. Heitor Vieira Dourado (FMT/HVD) in Manaus.

The drawing clearly represents the environment where the accident occurred, with emphasis on the fishing context and the natural elements of the region. At the center of the image is the canoe where the child and his brother were, floating on the water represented in blue. To the left of the scene, the sun indicates that the episode took place during the day. Close to the canoe, in the water, the aggressor snake is depicted in a central position and in exaggerated size.

The natural environment is detailed with green vegetation along the riverbanks, reinforcing the Amazonian and riverside setting of the location. Despite the care in depicting the environment, the drawing presents only one scene, with no sign of movement or representation of the journey to the hospital. The only means of transportation represented was the canoe; there is no depiction of the continuation of the route or of the medical care received.

The child does not depict himself in the image. The graphic style is classified as schematic, appropriate for his age group, with recognizable shapes, spatial organization, and expressive use of colors. The absence of human figures and other clinical developments (pain, injury, hospital, companions) highlights the focus on describing the immediate environment of the accident.
