# Supplementary material for: Therapeutic itineraries of children after snakebites in the Brazilian Amazon: A thematic drawing-and-story study
Source: PLoS Negl Trop Dis. 2025 Dec 1;19(12):e0013777. doi: 10.1371/journal.pntd.0013777 (PMC12677774; doi:10.1371/journal.pntd.0013777)
Supplement: Attachment — Submitted filename: Response to Reviewers ok.docx [file pntd.0013777.s007.docx]

**Dear Editor,**

We are grateful for the opportunity to review the manuscript titled *"PNTD-D-25-01184 -
Therapeutic Itineraries of Children after Snakebites in the Brazilian Amazon: a Thematic Drawing-and-Story Study"*. The reviewers' comments are very positive and will serve to improve the final version for publication, if accepted. Below, we present the responses to the reviewers, point by point.

**Reviewers' Comments:**

**Methods:**

Reviewer #1: This is a qualitative research study that includes quantitative clinical data and qualitative data from interviews that included drawing and narratives. The data collection tools (epidemiologic and TD-SP) are clearly described as are the methods used for managing and analyzing the data (transcription, descriptive statistics, coding, and content and thematic analysis). The study was overseen by an IRB committee and both parental and child participant acceptance was obtained.

Response: Thanks for the comments.

The following issues also need to be addressed:

- Please provide more detail on how participants were selected and recruited (e.g., was it every child presenting with snakebite during the study period?) and 2. how they were recruited for the study (e.g. a point person in the hospital alerted the study team who then approached the parent first and child second to explain the study and ask if they were willing to participate)? Clarify also whether all participants approached agreed to participate.
Response: Done accordingly.

- How was the sample size determined? If saturation was used how was this determined?
Response: Done accordingly.

- Re the first paragraph under ‘Data analysis’ in the methods section - It is not clear what this is about. How does "classification" relate to coding and theme identification described below? Consider clarifying this section, and if it does not add substantially to the analysis, it may be best removed.

Response: We revised the section to clarify that the descriptive analysis of the itineraries was based on the typology proposed by Cristino et al. (2024). We rewrote this part of the text for better understanding.

- Clarify in the last paragraph of the methods section if ‘categories’ are themes.
Response: Done accordingly.

- The methods section states that participants were told their drawings would not be shared yet these are included in the paper.

Response: Participants were assured that their identities would remain confidential, and that only their drawings and narratives would be disclosed without any identifying information. We rewrote this part of the text for better understanding.

Reviewer #2: This is a unique study and it is therefore not easy to simply answer some of these questions. This is a mixed-methods analysis of childrens' experiences with snakebite and seeking care for snakebite.

The study population is well-described. Not all components of the analysis are hypothesis driven, but I believe that is appropriate, as it is partially an exploratory and descriptive analysis.

Response: Thanks for the comments.

Reviewer #3: The objective of the study was to explore the therapeutic itineraries of 20 children following snakebites in the Brazilian Amazon, using a participatory approach based on their drawings and narratives. The study design is clearly stated; the population and data collection are described in detail. Methodological triangulation was used for robustness. Ethical approval has been obtained.

Response: Thanks for the comments.

- “No interviewer-related biases were identified”– how did you minimize/ check for interviewer bias?

Response: We rewrote this part of the text for better understanding, adding this information.

- Was member-checking performed?

Response: Member-checking was conducted during the interviews. At the end of each drawing activity, the child was asked to explain their therapeutic itinerary once again, and the researcher confirmed with the child whether this was indeed what they intended to express through the drawing and narrative.

- Potential power dynamics and cultural considerations should be addressed in the researcher’s reflexivity

Response: Done accordingly. This section was added to Research team and reflexivity.

- Please, add information on sample size, e.g. how was data saturation defined?
Response: Done accordingly.

- Please clarify how data was analyzed. In the abstract it is stated „Data was analyzed by deductive content analysis”. In the method section it’s stated that „A coding system was adapted from Goldner et al.“- applying deductive coding, and inductive coding was applied for narratives. Was a hybrid/ mixed approach used?

Response: We clarified this point in the Methods. The analysis of the drawings was descriptive, based on Goldner et al., focusing on style and content.

- „The results and methods report follow the Consolidated Criteria for Reporting Qualitative Research“ (S1 file). – The uploaded S1 file does not show COREQ guidelines, it shows Table 1. Characterization of the children who were victims of snakebites and participated in the study.

Response: Correction done accordingly.

**Results:**

Reviewer #1: Overall the results are presented in detail. The themes are especially well developed pulling from multiple sources and illustrated with quotes. In the first section (before themes) there is some repetition, and I think some of the detail can be reduced - more detail is included in my comments below.

- Details presented in Tables 2 and 5 can be moved to a supplement file with key examples extracted to illustrate main findings.
Response: Done as suggested.

- Table 2: P2 is “foot” an accurate translation? Based on context “boot” seems more likely.

Response: In this case, the child was indeed referring to the “foot,” and therefore the translation as “foot” is accurate.

And picking fruit in the tree is classified as Workplace for P2 and peridomestic for P4

Response: Table relocated as S3 File and clarified.

The classification reflects the specific context of each case. For P2, fruit picking occurred while also helping his father with fishing activities. In contrast, for P4, fruit picking occurred in his grandmother's backyard while playing with his sister.

- What is meant by active vs passive itineraries?

Response: Clarified in the text. In the Methods section, we clarified that the classification of therapeutic itineraries followed the explanatory model proposed by Cristino et al. (2024). According to this model, active itineraries are those in which the child makes decisions about their own therapeutic path, while passive itineraries are those in which decisions are made entirely by third parties (family members or healthcare professionals). Mixed itineraries are those in which both situations are observed in the same itinerary.

- Para starting “Table 3 presents the classification..” ends with this sentence “The following decisions were made by their companions.” However, this is not followed by any more information on the decisions made.

Response: Clarified accordingly.

- Para starting “Seventeen children…” states one child had mixed itinerary - and then goes on to give examples of 3 participants (P 5, 6 and 19) who seem to have mixed itineraries.

Response: Clarified accordingly.

- Para starting "Eighteen (90%) participants…” - what is the difference between ‘multidimensional’ and ‘fragmented’?

Response: Clarified accordingly. In our study, we followed the classification proposed by Cristino et al. (2024). Multidimensional itineraries refer to those in which children accessed different subsystems of care (folk, professional, or popular) in a complementary manner, with continuity across the trajectory. In contrast, fragmented itineraries are those characterized by interruptions or discontinuities, in which the child’s trajectory through the health system was marked by delays, incomplete referrals, or lack of follow-up.

- Para starting "Eighteen (90%) participants…”- There appears to be some repetition in this section. Consider streamlining by only briefly referring to Table 3 here and addressing the details more fully under themes

Response: Clarified accordingly.

- Table 3 would benefit from an additional column that explains the categories in more detail e.g. what is meant by ‘decision maker’? (decision on what?); what is fragmented vs continuous care?; What are other classification of severity?; if only 20% are public providers, what are the others?; what is meant by itinerary reporter?, etc
Response: We already have a published article presenting this classification in detail. Therefore, in this manuscript, we chose not to repeat the same descriptions to avoid redundancy and excessive detail. Instead, we directly reference the classification and guide the reader to consult Cristino et al. (2024), as indicated in the Methods section, for the complete information.

- Is there any information that can be shared on where the 4 participants who receive antivenom before being admitted to the hospital got it from?

Response: Clarified accordingly.

- ‘Drawing’ analysis section - Both the descriptive paragraph and the table communicate similar findings—consider retaining one and streamlining the other.

Response: Done accordingly.

Reviewer #2: The results do match the analysis plan.
The results are clear, but there is too much text and description. The results need to be better condensed and need to be presented in a much more concise manner. Things that the authors want to present that don't make it into the main paper can be presented in the supplemental files. As is, the paper reads more like a dissertation than a research paper. Figures are of sufficient quality and clarity.

Response: Part of the results were summarized, while the detailed information presented in Tables 2 and 5 was moved to the Supplementary Material. This allowed us to shorten the length of this section.

Reviewer #3: Overall, the presentation of the results could be improved. Reduce redundant information in both text and tables to improve clarity.

Response: Part of the results were summarized, while the detailed information presented in Tables 2 and 5 was moved to the Supplementary Material. This allowed us to shorten the length of this section.

The manuscript would benefit from including not only descriptive analysis of the data but also a critical interpretation of the drawings and narratives to provide deeper insight into the participants’ experiences.

Response: We acknowledge the value of a more critical interpretation of the drawings and narratives. However, in this study we intentionally limited our analysis to a descriptive level, as the scope was guided by the theoretical framework of therapeutic itineraries. We believe that a deeper interpretative approach to the drawings would require a distinct study specifically designed for that purpose. For the present manuscript, we therefore focused on a descriptive assessment, while suggesting future research dedicated exclusively to a critical evaluation of the drawings.

The topics of challenges in understanding the child’s narrative, perceptions of severity (e.g. hematuria), and health literacy, as reasons for delayed care-seeking are highly interesting.
Response: Thanks for the considerations.

Tables & Figures:
Include only necessary tables and figures in the main text; additional materials should be provided in the supplementary files.
Response: Done accordingly.

- e.g. Figure 3-7: are duplicates and are already included in the supplementary materials S2
Response: Correction were made accordingly. Drawings already described in the main text were removed from the Supplementary Material and are now presented in S5 File.

- Figure 2: Consider revising the data presentation format to enhance clarity and readability.

Response: Done accordingly.

- Table 3 and 5: reporting only descriptive data by individual participants may reduce readability and analytical value. Consider including critical analysis to highlight boarder patterns and insights.

Response: Done accordingly.

**Conclusions:**

Reviewer #1: The conclusions are clear and highlight the study's main findings. They are mostly well grounded in study findings. The sentences towards the end on future public health interventions, however, need to be supported by more references to evidence in existing literature (included in the main body of the discussion) to complement current study findings.

Response: Done accordingly.

Reviewer #2: The conclusions are supported by the data and also by other literature.
This is a small sample size, so it would be good to offer even more caution with regard to extrapolating to other similar individuals, let alone populations.

Response: We agree with the reviewer and have already addressed this point in the Limitations section at the end of the Discussion, highlighting the small sample size and the consequent caution required when extrapolating the findings to other individuals or populations.

There is some public health relevance here, and it is an important topic. I do believe a stronger public health argument could be made. There is clear public health relevance, but some of the text seems to get lost in social science theory rather than in practical, applied importance of the findings and what they might mean for public health etc.

Response: Thank you for the suggestion. We have revised the discussion to better emphasize the public health implications of our findings. At the same time, we chose to maintain the social sciences framework, as it is essential for understanding therapeutic itineraries not only as individual clinical trajectories, but also as sociocultural processes shaped by beliefs, relationships, and contexts. This theoretical grounding allows the practical public health relevance to be articulated in a way that is consistent with the lived experiences of participants and the complexity of care pathways.)

Do keep in mind that some aspects of what are being described are culture-specific and may also be symptoms of poverty. The authors note that such things must be taken into account by public health authorities. Children not being supervised, for example, might be quite relative across cultures. The paper almost tends toward casting blame for this when it could be culturally construed and/or a symptom of poverty.

Response: We agree that some aspects related to snakebite incidents in children may reflect cultural factors or be associated with poverty in the rural Amazonian context. Therefore, caution is needed to avoid interpretations that could be perceived as attributing blame to these factors. In response, we have revised both the discussion and the conclusion to make this perspective clearer.

Reviewer #3: Discussion
Consider reporting the key findings in the first paragraph of the discussion to provide context for the reader before interpreting them.
Response: Done accordingly.

“In the case of the children in this study, initial delays were often related to adults’ disbelief in the child’s account of the incident, difficulties in understanding the child’s narrative, and the consequent postponement of seeking professional care.” - This is a very important finding, how could this be improved? Are there any strategies or educational interventions in place for children in rural communities, who are at risk of snakebites?

Response: In the Amazonian rural context, there are currently no specific educational interventions or strategies in place targeting children who are at risk of snakebites. Following your suggestion, we added a paragraph to the discussion citing studies that recommend child-focused educational initiatives as a potential strategy to address this gap.

- Does Brazil have established guidelines for first aid and healthcare-seeking pathways following snakebite envenoming, and how do communities at risk obtain this information?
Response: Brazil has national guidelines for first aid and healthcare-seeking following snakebite envenoming; however, in remote Amazonian communities’ access to and dissemination of this information are limited. We have added this point to the conclusion.

Conclusion
In the abstract, the conclusion could be made more specific by highlighting how the findings might inform or enhance educational interventions for children and caregivers. Refining the abstract conclusion would also highlight the public health relevance of the study.

Response: Done accordingly.

**Editorial and Data Presentation Modifications?**

Reviewer #1: Introduction:

The introduction starts very clearly providing context on snakebites globally and in Brazil.

Response: Thank you for observation.

The concept of “healthcare itinerary" is critical to this study and required a clearer introduction and definition, with reference to literature on this approach. This could largely be achieved by revising the third paragraph.

Response: Clarified accordingly.

While interesting, too much historical information is provided on the history of the study of children’s perspectives - this would be better summarized in one paragraph that justifies why studying the child’s unique perspective is important.

Response: Thank you for this observation. We agree that the historical overview can be more concise, and we have revised it accordingly. However, we believe that retaining a brief historical context is important, as it helps to “denaturalize” the way children are viewed in contemporary society and justifies why it is essential to study their unique perspectives.

Methods:
- I find the paragraph that starts “In this study, a healthcare itinerary…” hard to follow and I am not sure what the purpose of having it in the methods section is.

Response: Clarified. In it we conceptualize the term healthcare itinerary used as one of the COREQ requirements.

- Consolidated Criteria for Reporting Qualitative Research should be cited- not attached as a supplement.
Response: Done accordingly (S1 File).

Discussion:
The discussion is currently the weakest element. It is overly long in parts and could be sharpened by focusing more directly on the key findings and situating them in the wider literature.

Response: Revised accordingly.

- The flow would be improved by starting with the key findings and setting it in context of the literature - and only then including one paragraph (not 5 or 6) on why this method is important - it will be evident at that point the added value that this approach has provided.

Response: Done accordingly.

- Each of the findings on the unique child child perspective identified in this study should be presented with a one sentence summary of the result and then setting it in context of the wider public health literature (beyond snakebites). E.g "child disbelief in adult account’ - how does this show up in studies on other public health issues? For the finding of a sense of trust around the house/ work you cite DooKeerem - has this phenomenon also been reported in other public health studies?; etc. This was done well for the finding ‘understanding child’s initial account’ where findings from Grills and Olendick were referenced.

Response: Clarified accordingly.

- How data showed up in the drawing is another important section of this discussion and substantial reference is appropriately made to the literature on this. That paragraph needs to start with one sentence summary of the relevant findings from this study - before unpacking and interpreting.
Response: Done accordingly.

- The in depth discussion on child labor while interesting is less relevant to this paper.

Response: Reduced and clarified accordingly.

Reviewer #2: There are some points that could be made more clear in the writing, but I believe the entire manuscript needs to be made vastly more concise before it is worth pointing out smaller grammatical edits, etc.

Response: Revised acccordingly.

**Summary and General Comments**

Reviewer #1: This is an important and novel study that uses detailed methods to illuminate children’s perspectives on snakebite care, with valuable implications for the design of public health interventions. At the same time, the manuscript is overly long in places, lacks focus in the discussion, and omits some key methodological details. Addressing these issues will substantially strengthen the clarity and impact of the paper.
Overall the writing is good but there are some sentences and paragraphs where the meaning is not clear to me. The paper would benefit from being reviewed by an editor.

Response: Thank you for your feedback. We have revised the manuscript for clarity, addressed the methodological details, and also had the text reviewed by a native English speaker.

Reviewer #2: I strongly suggest making the entire manuscript much more concise. There is background materials touching on the history of different social science approaches, etc. and much of this is both not sufficiently detailed to pass social science writing AND not really necessary for this public health journal. The writing also seems to have difficulty in its direction. Is this a methods piece, meant to showcase this style of triangulation? Or is this a study about snakebite and snakebite-related care among children? I don't believe it should be both, even though it could be a paper about snakebite and snakebite-related care, which showcases a novel approach to data collection and analysis (this is how I would frame this paper).

Response: Reduced and clarified accordingly.

The results also need to be condensed. Try to make one point = one paragraph at most, and then with only 1-2 quotes per point.

Response: Reduced and clarified accordingly.

Finally, in the discussion - keep this to being a paper about snakebite and snakebite related healthcare as a problem that needs to be addressed. What specifically did we learn? Is it similar or different from similar or very different settings? What are concrete public health things that we take from this. Try to be as concise as possible - this is different from normal social science writing. Most readers will not make it through something so long, and with as much materials about social science history and theory.

The goal should be to make this so that it is broadly accessible (including easy to read and get important points from it) - and perhaps especially for folks who might be in a position to deal with this problem.

Response: Done accordingly.

Reviewer #3: The manuscript contributes valuable insight into a neglected area of research. This study explores the therapeutic itineraries of 20 children following snakebites in the Brazilian Amazon, using a participatory approach based on their drawings and narratives combined with clinical and epidemiological data. The findings explore the children’s experience but also show how caregivers’ disbelief in the child’s account of the incident, perception of severity, and health literacy impact the delay in seeking professional care.

This study highlights the need to enhance health literacy in at-risk communities. This may influence timely care-seeking, adherence to first aid guidelines, and interpretation of children’s experiences following snakebite envenoming.

Response: Thank you for your feedback.

The presentation of the results could be improved. Reduce redundant information in both text and tables to improve clarity. The manuscript would benefit from including not only descriptive analysis of the data but also a critical interpretation of the drawings and narratives to provide deeper insight into the participants’ experiences.

Response: Thank you for this suggestion. In this study, we present only a descriptive evaluation of the drawings, as our main focus is on the healthcare itineraries. We agree that a more critical analysis of the drawings could provide additional insights, but we consider that such an in-depth approach would be better suited for a separate future study.

Cave: using participants numbers in citations and text may breach confidentiality, particularly in this small sample size and context-specific study. In addition, assigning participant numbers to detailed clinical data and drawings could compromise confidentiality (Supplementary Information 1. Table 1. Characterization of the children who were victims of snakebites and participated in the study; and Supplementary Information 2. Drawings of participants). The authors should clarify how data were anonymized or consider aggregating findings to prevent identification.

Response: Thank you for feedback. We ensured confidentiality by anonymizing all data and assigning only numerical identifiers to participants. Although the sample is small, the children live in geographically distant communities and do not share the same social circles, which minimizes the risk of identification. In qualitative research, even with small samples, the use of participant codes is a widely accepted practice to preserve anonymity while maintaining the richness of individual perspectives. For this reason, we consider that confidentiality is not compromised in our study.

Specific comments:

- Instead of the term “playful method”, the terms “child-friendly or age-appropriate method” could be used, this might be a more suitable term.

Response: Thank you for the suggestion. However, we opted to use the expression playful method because it is already widely employed in the literature to describe approaches that engage children through drawing and storytelling, and we cited authors who have previously used this terminology in similar studies.

- The term “fragmented transport” should be clarified.

Response: Done accordingly.

- “Through content analysis of the interviews with and drawings by participants,
five themes emerged” – please, improve sentence structure.

Response: Done accordingly.
